# Identifying excessive length of antibiotic treatment duration for hospital-acquired infections: a semi-automated approach to support antimicrobial stewardship

**DOI:** 10.1186/s13756-024-01406-4

**Published:** 2024-05-20

**Authors:** Suzanne M.E. Kuijpers, Koen J. van Haeringen, Thomas Groot, Kim C.E. Sigaloff, Reinier M. van Hest, Jan M. Prins, Rogier P. Schade

**Affiliations:** 1 Department of Internal Medicine, Division of Infectious Diseases, Amsterdam UMC, University of Amsterdam, Amsterdam, The Netherlands; 2 Department of Medical Microbiology and Infection Prevention, Amsterdam UMC, University of Amsterdam, Vrije Universiteit Amsterdam, Amsterdam, The Netherlands; 3 Department of Clinical Pharmacology, Department of Hospital Pharmacy, Amsterdam UMC, University of Amsterdam, Amsterdam, The Netherlands

## Abstract

**Background:**

Avoiding excessive antibiotic treatment duration is a fundamental goal in antimicrobial stewardship. Manual collection of data is a time-consuming process, but a semi-automated approach for data extraction has been shown feasible for community-acquired infections (CAI). Extraction of data however may be more challenging in hospital-acquired infections (HAI). The aim of this study is to explore whether semi-automated data extraction of treatment duration is also feasible and accurate for HAI.

**Methods:**

Data from a university-affiliated hospital over the period 1-6-2020 until 1-6-2022 was used for this study. From the Electronic Health Record, raw data on prescriptions, registered indications and admissions was extracted and processed to define treatment courses. In addition, clinical notes including prescription instructions were obtained for the purpose of validation. The derived treatment course was compared to the registered indication and the actual length of treatment (LOT) in the clinical notes in a random sample of 5.7% of treatment courses, to assess the accuracy of the data for both CAI and HAI.

**Results:**

Included were 10.564 treatment courses of which 73.1% were CAI and 26.8% HAI. The registered indication matched the diagnosis as recorded in the clinical notes in 79% of treatment courses (79.2% CAI, 78.5% HAI). Higher error rates were seen in urinary tract infections (UTIs) (29.0%) and respiratory tract infections (RTIs) (20.5%) compared to intra-abdominal infections (7.4%), or skin or soft tissue infections (11.1%), mainly due to incorrect specification of the type of UTI or RTI. The LOT was accurately extracted in 98.5% of courses (CAI 98.2%, HAI 99.3%) when compared to prescriptions in the EHR. In 21% of cases however the LOT did not match with the clinical notes, mainly if patients received treatment from other health care providers preceding or following the present course.

**Conclusion:**

Semi-automatic data extraction can yield reliable information about the indication and LOT in treatment courses of hospitalized patients, for both HAI and CAI. This can provide stewardship programs with a surveillance tool for all in-hospital treated infections, which can be used to achieve stewardship goals.

**Supplementary Information:**

The online version contains supplementary material available at 10.1186/s13756-024-01406-4.

## Background

Antimicrobial stewardship programs (ASPs) have been developed to measure and improve antimicrobial prescribing, aiming to optimize the use of antibiotic and other antimicrobial agents [1–4]. One of the key quality indicators of antimicrobial stewardship (AMS) is the adherence to local or national antimicrobial guidelines with regard to choice of antibiotic and treatment duration [5]. To monitor prescribing practices and assess the effectiveness of antimicrobial stewardship initiatives, a continuous process of gathering structured data is essential. Traditionally, manual data extraction from Electronic Health Records (EHRs) was necessary to gain insight into prescribing trends in a healthcare setting, for instance by means of a point-prevalence survey. This is a time-consuming process. Nowadays, most software programs in which electronic patient records are managed support obtaining this information on a semi-automatic basis. This approach allows for the efficient gathering of large amounts of structured data on antimicrobial prescriptions. This not only enables tracking of trends in antimicrobial use within a hospital, but also enables comparison of antimicrobial use and benchmarking between different healthcare facilities.

**Fig. 1:**
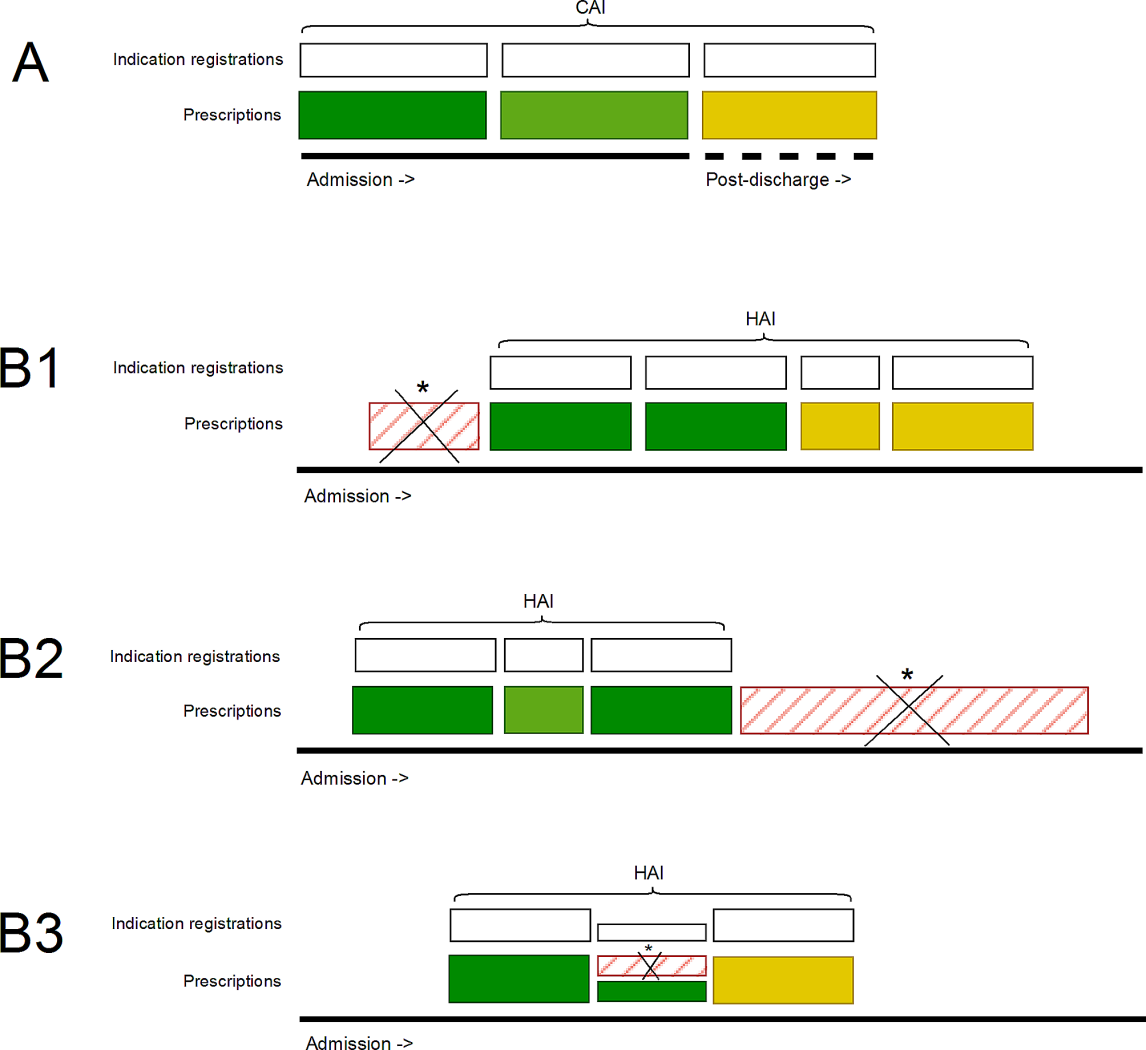
Differences in data-complexity in community-acquired infections versus hospital-acquired infections. (**A**) Treatment courses in community-acquired infections (CAI) usually consist of sequential building blocks that can be easily assembled. (**B1**) Treatment courses in already hospitalized patients are often more complex and need to be thoroughly filtered to remove non-related prescriptions. Examples: B1. A prescription registered as prophylaxis has to be removed to prevent inclusion as starting point of the treatment course. (**B2**). An unlabelled prescription directly initiated after treatment has to be removed as it is a prophylactic prescription (duration > 28 days). B3. A non-related prophylactic prescription but started during HAI treatment has to be removed. Coloured blocks represent different types of antibiotics e.g. meropenem, ceftriaxone, ciprofloxacin

To be able to analyze trends in guideline adherence, including the duration of treatment, the indication for the antibiotic prescriptions is crucial information. Previously published literature demonstrates that healthcare provider-selected indication tools yield reliable information [6–8]. We previously showed that it is feasible to take data from a mandatory indication registration and use dedicated scripting to combine it with other raw data in the EHR, to extract the total length of treatment (LOT) for community-acquired infections (CAI) [9]. Scripts that are primarily based on the concatenation of sequential data blocks however cannot be used for processing data on hospital acquired infections (HAI). In patients that are already admitted, data on antibiotic prescriptions are generally far more complex and need dedicated and elaborate processing of data (Fig. 1). This can be explained by the fact that these infections are usually intercurrent events among admitted patients with multiple clinical problems. Furthermore, also complicating factors such as the presence of multiple infections and prophylactic use of antibiotics make semi-automatic surveillance of antibiotic treatment duration of HAI more complex.

The primary objective of this study is to analyze whether it is possible to extract reliable data on treatment of HAI in addition to CAI, and whether this method can yield accurate information on the treatment duration of HAI, for use in semi-automated surveillance.

## Methods

### Study design and setting

A retrospective study was conducted using data from the Amsterdam University Medical Centers Amsterdam (Amsterdam UMC), a university-affiliated tertiary care hospital. This institution uses Epic (Epic Systems Corporation) as its EHR and prescription software. For all in-hospital antimicrobial prescriptions, a mandatory indication registration tool is used to document the specific indication for which the antimicrobial agent is prescribed [8, 9]. Approval from the institutional review board for this study was not required because retrospective, pseudonymized data was used for quality optimization purposes. Procedures were in accordance with the General Data Protection Regulation [10].

### Data collection and definitions

Data was extracted from the period of 1-6-2020 to 1-6-2022. The following data was extracted: prescriptions with an antimicrobial agent belonging to the Anatomical Therapeutic Chemical (ATC) class J01, corresponding indications linked to these prescriptions, and hospital admission information (admission and discharge date). The possible indications were empirical therapy, targeted therapy or prophylaxis. In case of therapy, the prescriber had to select the main focus of infection, first on tract level, followed by a further specification for cases involving urinary tract infections (UTI) and respiratory tract infections (RTI) [8, 9] (additional file 1).

**Fig. 2:**
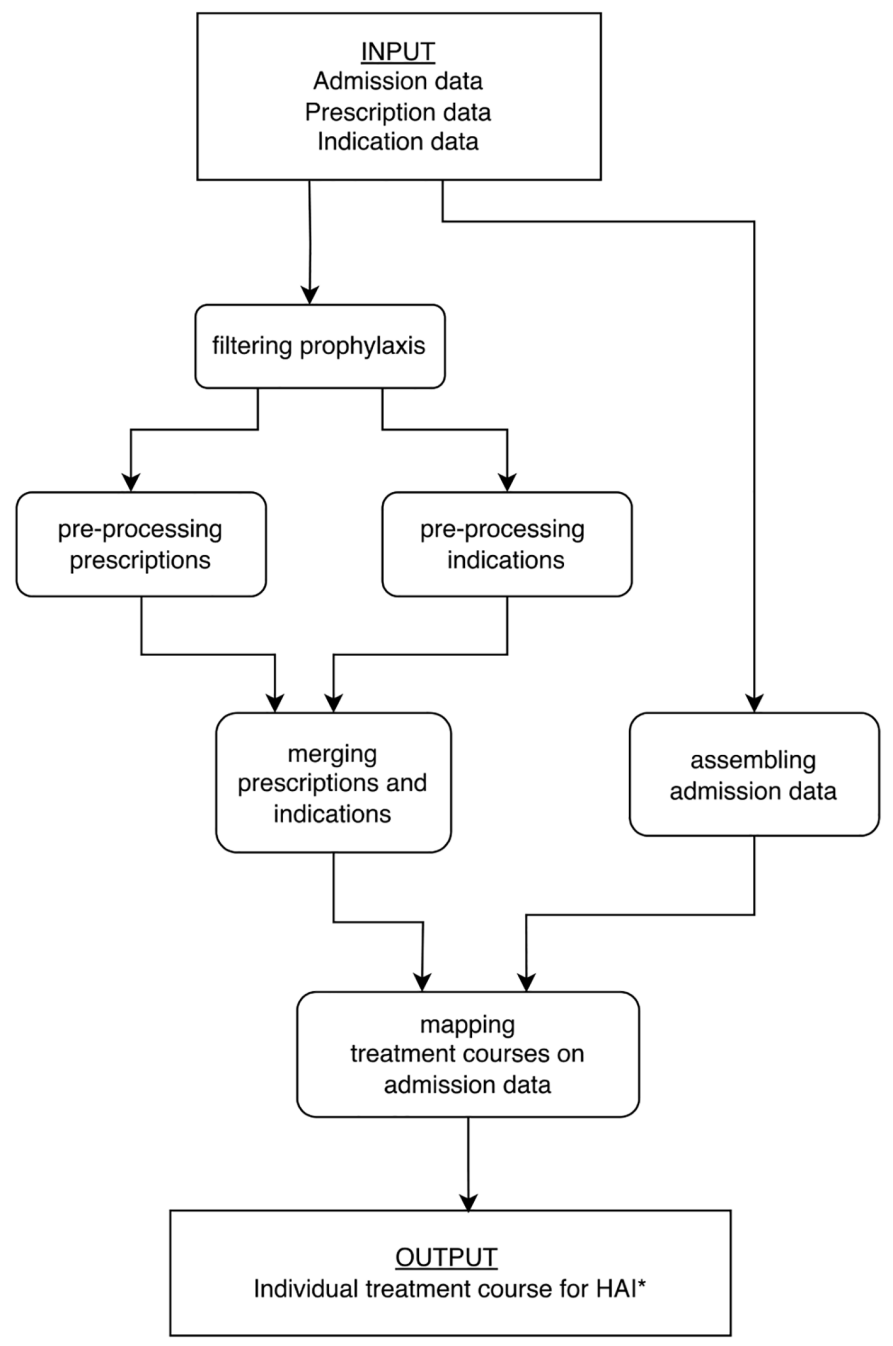
Data flow diagram. The code takes raw data as input, and produces for each patient an individual treatment course. *Metadata include: patient_ID, final diagnosis, start and stop date, total duration, names of used antibiotics

Raw data from the EHR including antibiotic prescriptions and corresponding indications were filtered and assembled by using a newly designed, dedicated python-script. The data flow during the processing of the raw data is shown in Fig. 2. In short, all therapeutic antibiotic prescriptions initiated after the start of an antibiotic treatment and within a maximum interval of 24 h after a prior stop date were linked and transformed into treatment courses, including outpatient prescriptions during the post-discharge period. The first registered indication was defined as the start of the treatment course. The last registered indication within a treatment course was labeled as the definitive indication. Additional file 2 shows a detailed pseudo-code of the python-script.

For filtering of presumed prophylaxis, the following antimicrobials were considered to be prophylactic if prescribed for more than 28 days: azithromycin, clarithromycin, doxycycline, erythromycin, fosfomycin, nitrofurantoin, pheneticillin, and trimethoprim. In addition, prescriptions of trimethoprim/sulfamethoxazole were deemed prophylactic if prescribed in low dosages (480 mg or lower) for a duration of more than 14 days and in higher dosages for a duration of more than 50 days. The LOT was defined as the number of calendar days during which antimicrobials were consecutively prescribed for the definitive indication, including post-discharge duration, irrespective of the number of agents or doses on each calendar day.

We included all data on treatment courses which were initiated either in the ER or during hospitalization. For this study, hospitalization was defined as a minimum admission duration of 12 h. An infection was defined as community-acquired (CA) when antibiotic treatment was started less than 48 h from admission and hospital-acquired (HA) when antibiotic treatment was started later than 48 h from admission. We excluded all treatment courses in pediatric patients and patients that had been admitted to the intensive care unit (ICU) during admission. Finally, we excluded courses in which multiple diagnoses were registered as final diagnosis or in which the indication was entered in an open text field (additional file 1). The collected data partly overlaps with data used in an earlier study, to be able to compare results of the treatment duration of CAI and HAI. All data were re-analyzed using the new script.

### Validation of the dataset

To validate the retrieved dataset a random sample was generated for a manual check. For UTI, RTI, and intra-abdominal infection (IAI) a random sample of 5% per infection was chosen. Of the remaining diagnoses another 5% sample was validated. After initial general inspection of the data a higher error rate was expected for courses with an extended duration (more than 45 days) due to prescriptions that were incorrectly not stopped. Therefore, 20% of these courses was validated. This resulted in the validation of 5.7% of the total dataset. Validation of the indication consisted of comparison of the indication registered in the prescription with the diagnosis in the clinical notes in the EHR. To validate the treatment duration, courses were evaluated in two steps. First, the calculated treatment duration was compared to the duration based on prescriptions in the EHR. This was performed to validate whether the code reliably calculated the length of treatment (LOT) from the extracted prescriptions. Second, the calculated duration of treatment was compared to the clinical notes and discharge papers. This was done to identify factors that are not incorporated in the code and could lead to an incorrect LOT. Examples of such factors are transfers from and to other facilities, in which case data on the start or end of treatment misses in the EHR prescription information. Validation of the dataset was manually performed by one researcher (SK). When uncertain, a second (RS) and third researcher (JP) were consulted.

### Study endpoints

The primary endpoint of this study was the accuracy of the retrieved indications and calculated LOT of the validated sample of treatment courses. The accuracy was defined as the error rate of the semi-automatic compared to the manually retrieved indications and LOT. The secondary endpoint was the difference in antibiotic treatment duration between CAI and HAI.

### Data analysis

As a low accuracy was expected for treatment courses of more than 45 days and for treatment courses during which a patient was readmitted, these were analyzed first. For the validated courses, the error rate for indications and LOT were presented as percentage of the total number of validated courses for that indication. This was calculated by dividing the incorrect retrieved indication or calculated LOT by the total number of validated courses. Next, the median LOT with interquartile range and the percentage of post-discharge treatment were analyzed per indication for both CAI and HAI. All data handling and visualization was performed using TIBCO Spotfire.

## Results

**Fig. 3:**
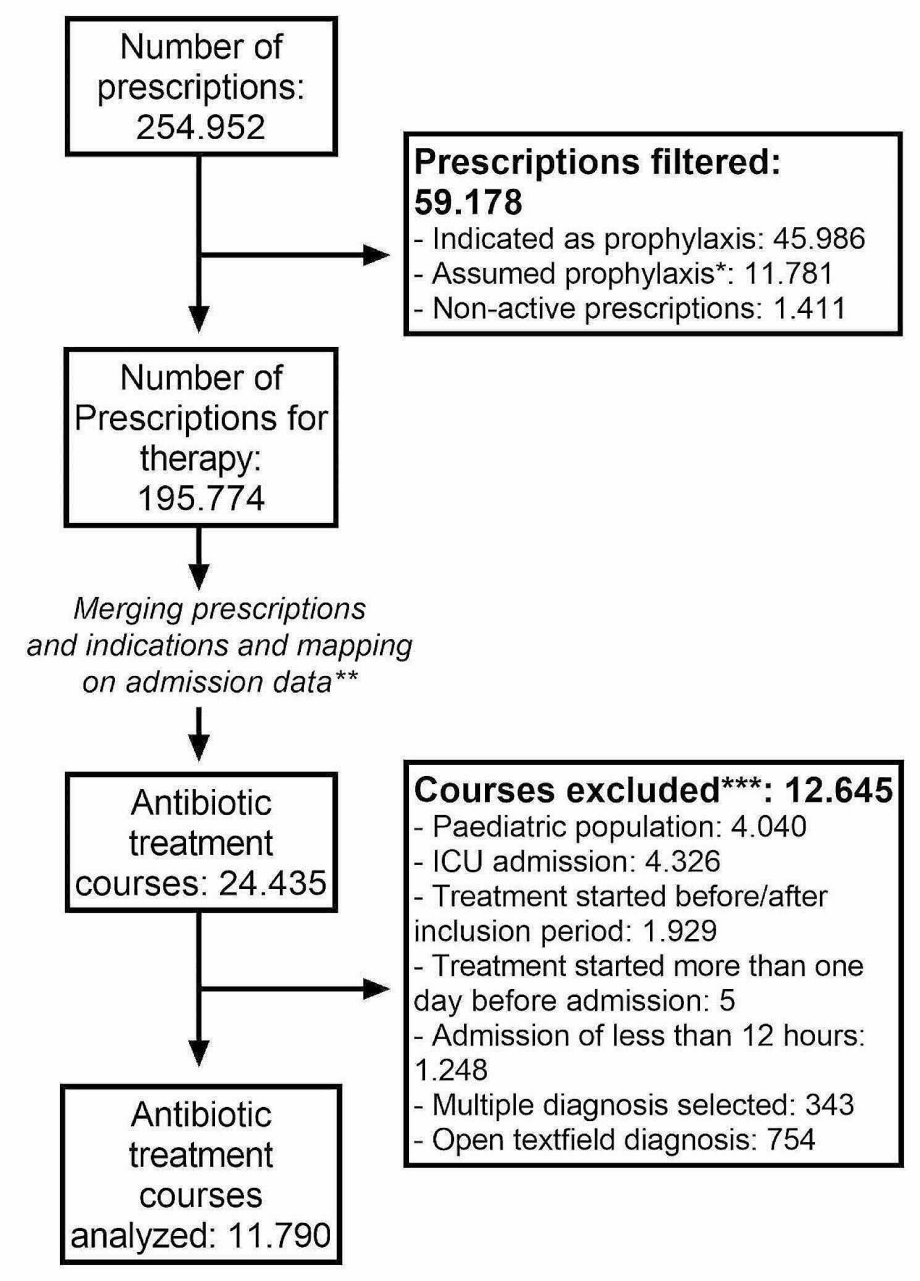
Flow diagram showing the data selection steps resulting in the final dataset. *See Methods. **Consecutively prescribed antibiotics during hospital admission and post-discharge. ***In order of exclusion, exclusion criteria might overlap

After applying the script to the raw data, the code produced a total of 24.435 treatment courses. After applying our predefined in- and exclusion criteria, 11.790 courses remained for further analysis (Fig. 3). Of these courses a random sample of 673 courses was selected for validation: 144 UTI, 118 RTI, 87 IAI, 219 various other indications, and 105 courses with an extended duration (> 45 days). First, treatment courses of more than 45 days and treatment courses of patients readmitted within the treatment course were analyzed. In these treatment courses, over 50% of the calculated LOT did not represent the clinical course as documented in the EHR. We decided to exclude these treatment courses from further analyses. A total of 10.564 treatment courses remained. 73.1% of infections were labeled as CAI and 26.8% as HAI. The distribution of infections over these two groups can be found in Table 1. Some infections such as cystitis and febrile neutropenia were more frequently encountered as HAI. Cystitis was among the most commonly diagnosed infections during hospital admission.

**Table 1:** Distribution of community- and hospital-acquired infections, with their length of treatment

	Community-acquired, *n* (% of total CAI)	LOT in days, [median; IQR]	Hospital-acquired, *n* (% of total HAI)	LOT in days, [median; IQR]	Total, *n* (% of total infections)
Intra-abdominal infection *	1191 (15.4)	6.0 [4.0–9.0]	435 (15.3)	6.0 [4.0–10.0]	1626 (15.4)
**RTI-CAP-m**	672 (8.7)	6.0 [4.0–8.0]	71 (2.5)	6.0 [4.0–7.0]	743 (7.0)
**RTI-CAP-s**	215 (2.8)	5.0 [3.0–7.0]	23 (0.8)	5.0 [3.5-6.0]	238 (2.3)
**RTI-HAP**	126 (1.6)	6.0 [4.0–8.0]	234 (8.3)	6.0 [4.0–8.0]	360 (3.4)
**RTI-COPD**	147 (1.9)	7.0 [3.0–8.0]	33 (1.2)	6.0 [3.0–8.0]	180 (1.7)
**RTI-Aspiration**	175 (2.3)	7.0 [3.0–9.0]	88 (3.1)	7.0 [5.0–9.0]	263 (2.5)
**RTI-abscess/empyema**	50 (0.6)	14.5 [6.3–24.8]	14 (0.5)	33.0 [16.8–41.5]	64 (0.6)
**RTI-other (not specified)**	302 (3.9)	6.0 [3.0–9.0]	72 (2.5)	6.5 [4.8–8.3]	374 (3.5)
**UTI-cystitis**	845 (10.9)	6.0 [2.0–9.0]	505 (17.8)	6.0 [4.0–8.0]	1350 (12.8)
**UTI-complicated**	544 (7.0)	11.0 [5.0–15.0]	106 (3.7)	11.0 [6.0–15.0]	650 (6.2)
**UTI-kidney transplant**	144 (1.9)	14.0 [7.0–15.0]	40 (1.4)	11.0 [4.8–15.3]	184 (1.7)
**UTI-Catheter- related**	87 (1.1)	9.0 [3.0–15.0]	64 (2.3)	7.0 [4.0–11.0]	151 (1.4)
**UTI-other (not specified)**	193 (2.5)	8.0 [3.0–14.0]	42 (1.5)	6.5 [2.0-11.8]	235 (2.2)
**Bone or joint infection**	470 (6.1)	8.0 [2.0–16.0]	97 (3.4)	8.0 [3.0–16.0]	567 (5.4)
**CNS infection**	112 (1.4)	7.0 [3.0-14.3]	35 (1.2)	8.0 [6.5–12.5]	147 (1.4)
**E.N.T. or oral and maxillofacial**	212 (2.7)	10.0 [7.0–13.0]	90 (3.2)	9.0 [6.0–11.0]	302 (2.9)
**Febrile neutropenia**	59 (0.8)	5.0 [3.0-6.5]	123 (4.3)	4.0 [4.0–6.0]	182 (1.7)
**Gastro-enteritis**	105 (1.4)	5.0 [3.0–9.0]	39 (1.4)	6.0 [3.0-9.5]	144 (1.4)
**Gynaecological infection**	377 (4.9)	2.0 [1.0–8.0]	78 (2.8)	2.0 [1.0–5.0]	455 (4.3)
**CVL infection**	108 (1.4)	6.0 [3.0–11.0]	74 (2.6)	5.0 [3.0–8.0]	182 (1.7)
**Mediastinitis**	48 (0.6)	8.0 [3.0–15.0]	14 (0.5)	8.5 [7.0-14.8]	62 (0.6)
**SAB**	67 (0.9)	13.0 [8.00-18.5]	22 (0.8)	15.5 [12.0–16.0]	89 (0.8)
**Sepsis e.c.i.**	717 (9.3)	4.0 [2.0–7.0]	319 (11.3)	5.0 [3.0–7.0]	1036 (9.8)
**Skin or soft tissue infection**	672 (8.7)	11.00 [6.0–15.0]	195 (6.9)	10.0 [6.0–13.0]	867 (8.2)
Not further specified*	91 (1.2)		22 (0.8)		113 (1.1)
**Total**	7729	6.0 [3.0–7.0]	2835	6.0 [4.0–10.0]	10,564

* Numbers are n(%)

** Indications rarely chosen (less than 50 times in dataset)

CAI = community-acquired infection, HAI = hospital-acquired infection, LOT = length of treatment, RTI = respiratory tract infection, CAP-m = mild-to-moderate severe community-acquired pneumonia (PSI 1–2), CAP-s = severe community- acquired pneumonia (PSI 3–5), HAP = hospital-acquired pneumonia, COPD = chronic obstructive pulmonary disease, UTI = urinary tract infection, CNS = central nervous system, E.N.T. = ear, nose, and throat, CVL = central venous line, SAB = *Staphylococcus aureus* bacteremia

### Validation of indication

After excluding 105 courses with extended duration and 97 courses of patients readmitted (exclusion may overlap), the remaining 529 courses were analyzed (Table 2; broken down per infection in additional file 3). As some of the validated treatment courses within the validated sample of UTI, RTI, IAI or other infections were also of extended duration or in readmitted patients these were also excluded. The error rate of extracted indications was comparable with the error rates we found in an earlier study [9]. In 21.0% of courses the extracted indication did not match the indication of the treatment course found in the EHR. This decreased to 12.3% if results were analyzed based on site of infection (without further specification of type of UTI or RTI). 44.2% of registered sepsis e causa ignota (e.c.i.) indications were incorrect. In 20.8% of these labeled sepsis e.c.i. courses the right diagnosis was febrile neutropenia after analysis of the clinical notes. Other frequent errors in indications were the selection of cystitis instead of complicated UTI or selection of severe CAP instead of mild CAP, or vice versa (additional file 3).

**Table 2:** Error rate in validated sample of antibiotic courses

	Incorrect registered indication, *n* (%)	Incorrect LOT based on prescriptions, *n* (%)	Incorrect LOT based on clinical notes, *n* (%)
**Intra-abdominal infections**	6/84 (7.4)	2/84 (2.4)	22/84 (26.2)
Respiratory tract infections (specified)*	23/112 (20.5)	2/112 (1.8)	17/112 (15.2)
Urinary tract infections (specified)*	38/131 (29.0)	0/131 (0.0)	30/131 (22.9)
**Total of other infections**	44/202 (21.8)	4/202 (2.0)	42/202 (20.8)
**Total of validated infections**	111/529 (21.0)	8/529 (1.5)	111/529 (21.0)

*Only assigned as correct when the type of respiratory tract infection or urinary tract infection was specified, e.g. community-acquired pneumonia-m, abscess/empyema, or urinary tract infection – complicated

LOT = length of treatment

### Validation of LOT

In only 8 courses (1.5%) the LOT calculated using our code differed from the LOT based on the prescriptions in the EHR (Table 2 and additional file 3). This was usually the case when electronic prescriptions incorrectly had no stopping date. When this was the case, the code would stop the treatment course on the start date of the last prescription. Alternatively, these prescriptions without stopping date were manually stopped at a later outpatient visit or when the patient was readmitted. This would lead to too long treatment courses. The latter courses were mostly discarded because we excluded treatment courses of more than 45 days.

During the second validation step the data was validated based on the treatment duration registered in the clinical notes. The calculated LOT differed from the clinical notes in the EHR in 21.0% of courses. In 37.6% of these cases this was due to transfer from or to another hospital, leading to incorrect treatment durations using the code. In 14.1% treatment had already started before admission (e.g. by a general practitioner) and was continued during admission. In 10.6%, errors were due to antibiotics incorrectly labelled as prophylaxis, and therefore not included in our code. Another 10.6% of errors were attributable to patients receiving outpatient parenteral antimicrobial therapy (OPAT). As these prescriptions are not yet electronically available, these are not registered as prescriptions in the EHR. Finally, 4.7% of errors were due to vancomycin prescriptions, which are often prescribed as single doses, sometimes given more than 24 h apart. The remaining 22.4% of errors could be attributed to several different reasons.

Third, we compared the accuracy between CAI and HAI (Table 3). We did not find a difference between accuracy of extracted indications or accuracy of LOT based on the prescriptions in the EHR. However, the accuracy of LOT based on the clinical notes was lower in hospital-acquired infections.

**Table 3:** Error rate in community- versus hospital-acquired infections

	Incorrect registered indication, *n* (%)	Incorrect LOT based on prescriptions, *n* (%)	Incorrect LOT based on clinical notes, *n* (%)
**Community-acquired infections**	79/380 (20.8)	7/380 (1.8)	89/380 (23.4)
**Hospital-acquired infections**	32/149 (21.5)	1/149 (0.7)	22/149 (14.8)
**Total**	111/529 (21.0)	8/529 (1.5)	111/529 (21.0)

LOT = length of treatment

### Duration of treatment for CAI and HAI

Table 1 shows the distribution of infections for CAI and HAI, with their median calculated LOT. The LOT did not differ between CAI and HAI. It must be noted that infections that require a long treatment duration have a lower median treatment duration than expected due to the exclusion of courses of more than 45 days (e.g. bone or joint infections and *Staphylococcal aureus* bacteremia). Of all treatment courses of CAI 37.5% received part of the treatment post-discharge, compared to 23.7% of HAI. If part of the treatment was given post-discharge, this percentage of the total treatment duration was similar for CAI (59.9%) and HAI (56.7%).

## Discussion

This study demonstrates the accuracy and reliability of semi-automatic extraction of data on treatment duration for HAI, for both the in-hospital and post-discharge treatment period. In approximately 20% of treatment courses the indication was incorrectly registered. The code could effectively extract prescriptions from the EHR and transform data into treatment courses, but this did not resemble clinical practice in approximately 20% of cases, mainly when patients received treatment from other health care providers preceding or following the present course. The semi-automatic gathering of data allows antimicrobial stewardship teams to easily identify areas for improvement and to measure the effect of interventions, for both HAI and CAI.

For a number of categories the method of semi-automatic data extraction turned out to be not reliable. Treatment courses exceeding 45 days had a low accuracy. One potential reason for the lower accuracy could be that the longer a treatment course lasts, the greater the likelihood of incorrect prescriptions, for example due to incorrectly not stopping prescriptions or labelling treatment incorrectly as prophylaxis. Another category with a high error rate were treatment courses in which the patient was readmitted before the end of the treatment course. Therefore these treatment courses were excluded from further analyses.

In 79.0% of treatment courses the extracted indications matched the data found in the clinical notes. The main exceptions were the incorrect use of moderate versus severe CAP and cystitis versus complicated UTI. The error rates for these indications warrant caution when interpreting the results and ask for more awareness with clinicians to correctly enter indications. The calculated LOT by using our code differed from the prescriptions in the EHR in only 1.5%. However, in 21.0% the calculated duration of therapy was not in compliance with the clinical notes. This discrepancy could be largely attributed to patients admitted from and transferred to another hospital, already initiated therapy before admission, and OPAT prescriptions. These factors remain a challenge in semi-automatic surveillance of this data.

The results of the entire dataset showed that there were no large differences in treatment duration for CAI and HAI. When considering only courses with post-discharge treatment, we observed that a similar proportion of treatment courses was given post-discharge. Our previous study showed that many infections are still treated too long and a considerable amount of this excessive treatment is given post-discharge [9]. Possible stewardship interventions could focus on antibiotic overuse at discharge for both CAI and HAI. These interventions focused on post-discharge prescriptions have shown to be effective in reducing incorrect antibiotic exposure [11, 12].

Several studies have shown that it is feasible to use data from the EHR for antimicrobial stewardship purposes [8, 9, 13, 14]. Monitoring treatment duration including post-discharge prescriptions has not been widely studied before. Data about the reliability of the LOT in CAI, including post-discharge prescriptions, has been studied before by our group in the same hospital [9]. In this previous study all antibiotic treatment courses initiated more than 24 h after admission were excluded. In the current analysis these were included, generating also an overview of HAI [9]. Furthermore, the treatment courses were not only compared to the prescriptions in the EHR but also to clinicians’ notes. Errors discovered in this category pose a greater challenge when it comes to integration into the code, as it requires the extraction of more complex variables. The data should lead to stewardship interventions aiming at more guideline-compliant antibiotic prescribing as many treatment courses are still prescribed too long, with all its undesirable consequences [9, 15].

Semi-automatically gathered data also holds the potential to be used for monitoring of other antimicrobial stewardship objectives. For example, data containing information about whether cultures are taken and the results of these cultures could be added to each course, to examine appropriate de-escalation of therapy or the timely switch from intravenous to oral therapy [3]. Renggli et al. have recently shown the wide variety of stewardship indicators that could be monitored with data from the EHR [13]. Sharing this data between different healthcare institutions allows benchmarking of antimicrobial use.

### Strengths and limitations

A major strength of the design of this study is the use of already stored data from the EHR leading to a sustainable method of surveillance. This data was extracted from a large hospital with a large population, enabling evaluation of a large number of antibiotic courses for different indications. As this study investigates both community- and hospital-acquired infections it gives a broad overview of the quality of antimicrobial use.

A limitation of this study is that we used data extracted from proprietary EHR-software by Epic (Epic Systems Corporation). Van den Broek et al. already showed that extracting registered indications from Chipsoft software is possible and results in comparable useful stewardship information [8]. However, not many hospitals use an indication registration tool as it requires additional registration for clinicians. Without the use of this tool it is difficult to assign an indication to a treatment course limiting the usefulness for antimicrobial stewardship interventions. Another limitation of this type of monitoring is that the reliability of data depends on what indication clinicians enter, in our study leading to a 20% error rate in chosen indications. Recent studies have shown that this error rate is acceptable [7–9]. However, interventions should take place to decrease this error rate.

## Conclusion

This study shows that semi-automated extracted data can be used for monitoring treatment duration of both CAI and HAI, by generating a detailed overview of all in-hospital treated infections. When excluding long treatment courses and readmissions, the extracted data can provide stewardship programs with a surveillance tool to initiate and monitor stewardship projects aiming at improving guideline adherence, including the duration of treatment.

### Electronic supplementary material

Below is the link to the electronic supplementary material.

Supplementary Material 1: Additional file 1: Electronic medication registration prescription format; Additional file 2: Pseudo-code of the extraction-script; Additional file 3: Error rate in validated sample of courses, broken down per infection.

## Data Availability

The dataset used and/or analyzed during the current study are available from the corresponding author on reasonable request. The (pseudo-)code/syntax used for this study is available on reasonable request.

## Abbreviations

ASP: Antimicrobial stewardship programs
AMS: Antimicrobial stewardship
EHR: Electronic Health Records
LOT: Length of treatment
CAI: Community-acquired infection
HAI: Hospital-acquired infection
ATC: Anatomical Therapeutic Chemical
UTI: Urinary tract infection
RTI: Respiratory tract infection
ER: Emergency room
CA: Community-acquired
HA: Hospital-acquired
IAI: Intra-abdominal infection
ICU: Intensive care unit
OPAT: Outpatient Parenteral Antimicrobial Team
