# Supplementary material for: Identifying excessive length of antibiotic treatment duration for hospital-acquired infections: a semi-automated approach to support antimicrobial stewardship
Source: Antimicrob Resist Infect Control. 2024 May 20;13:52. doi: 10.1186/s13756-024-01406-4 (PMC11103818; doi:10.1186/s13756-024-01406-4)
Supplement: Supplementary file 1 — Supplementary Material 1: Additional file 1: Electronic medication registration prescription format; Additional file 2: Pseudo-code of the extraction-script; Additional file 3: Error rate in validated sample of courses, broken down per infection. [file 13756_2024_1406_MOESM1_ESM.docx]

**Additional files**

**Additional file 1. Electronic medication registration prescription format**

| 1. Select antimicrobial agent | |
| --- | --- |
| 2. Select indication | □ Empirical therapy □ Targeted therapy □ Prophylaxis □ IV-oral switch |
| 3. Select focus of infection | □ Bones and joints □ Central nervous system □ Gastrointestinal □ Yeast/mycosis  □ Gynaecological infection □ Skin and soft tissue □ Intra-abdominal infection □ Ear-nose- and throat infection □ Febrile neutropenia □ CVL infection □ Respiratory tract infection □ Mediastinum □ Eye □ Staphylococcus aureus bacteraemia □ Urinary tract infection  □ Unknown/sepsis of unknown cause □ Other |
| 4. Select specified indication | |
| *In case of Respiratory tract infection* | □ Bronchitis- exacerbation of COPD □ Community acquired pneumonia – severe  □ Community acquired pneumonia mild to moderate severe □ Aspiration pneumonia  □ Hospital acquired pneumonia □ Lung abscess/pleural empyema □ Other |
| *In case of Urinary tract infection* | □ Cystitis □ Prostatitis, chronic □ Pyelonephritis □ Catheter-associated urinary tract infection □ Polycystic kidney urinary tract infection □ Urosepsis  □ Urinary tract infection in renal transplant |
| *In case of Febrile neutropenia* | □ Low-risk neutropenia □ High-risk neutropenia □ Not applicable |

Reprinted from van den Broek et al. Detecting inappropriate duration of antimicrobial therapy using semi-automated surveillance. Antimicrobial Resistance and Infection control. 2022; 11(1):110. Reprinted with permission. IV = intravenous, CVL = central venous line, COPD = chronic obstructive pulmonary disease.

**Additional file 2. Pseudo-code of the extraction-script**

import pandas as pd

from datetime import timedelta

# Max duration for merging voorschiften in DAYS

max_duration = 1

# 10 minutes duration

minutes_10 = timedelta(seconds=600)

# Find relevant voorschriften

voorschriften = set()

for row in antibiotica_orderid:

voorschriften.add(row)

og_vs_dict = {}

for vid, start, stop, ab, pid, orderstatus in zip(voorschrift_voorschriftid,

voorschrift_startdatumtijd,

voorschrift_stopdatumtijd,

voorschrift_sysab,

voorschrift_pseudo_id,

voorschrift_orderstatus):

# Skip wrong values

if vid is None or vid == "" or ab is None or ab == "":

continue

# skip missing dates

if start is None or stop is None: continue

# Correction for single gift

if start == stop:

stop = stop + minutes_10

# Remove incorrect values and cancelled voorschriften

if stop < start or orderstatus == "Geannuleerd": continue

# Create id entry in dict

if pid not in og_vs_dict:

og_vs_dict[pid] = {}

# create ab entry in dict

if ab not in og_vs_dict[pid]:

og_vs_dict[pid][ab] = []

# add voorschriften data to dictionary

og_vs_dict[pid][ab].append([start, stop, vid])

ab_data = {}

# For every diagnosis / question / answer

for pid, id, vraag, antwoord, orderstart, orderstop in zip(antibiotica_pseudo_id,

antibiotica_orderid,

antibiotica_orderspecificatievraagformulering,

antibiotica_orderspecificatievraagantwoord,

antibiotica_startdatumtijd,

antibiotica_stopdatumtijd):

# replace , to prevent issues with csv files

vraag = vraag.replace(",", "/")

antwoord = antwoord.replace(",", "/")

# skip orders without start or stop date

if orderstart is None or orderstop is None: continue

# add new row if pid is not present in data

if pid not in ab_data:

ab_data[pid] = []

# add data to corresponding row

ab_data[pid].append([orderstart, id, vraag, antwoord, orderstop])

print("ab_data: ", len(ab_data))

# sort diagnoses

for pid in ab_data:

ab_data[pid] = sorted(ab_data[pid])

print("ab_data: ", len(ab_data))

# Merge voorschriften for every diagnosis

single_day = timedelta(1)

matched_all_vs = []

# For every patient with a diagnosis

for pid in og_vs_dict:

# for all ab given to that patient

for ab in og_vs_dict[pid]:

if pid not in ab_data: continue

# for all diagnoses for that patient

for diag in ab_data[pid]:

# Filter diagnoses with no voorschriften

id = diag[1]

if id not in [vs[2] for vs in og_vs_dict[pid][ab]]: continue

# Get diagnosis info

orderstart = diag[0]

vraag = diag[2]

antwoord = diag[3]

orderstop = diag[4]

lange_voorschrift = 0

start_stop_times = [orderstart, orderstop, [id]]

# merge all relevant voorschriften that overlap with diagnosis

for vs in sorted(og_vs_dict[pid][ab]):

start = vs[0]

stop = vs[1]

vid = vs[2]

# skip voorschriften before any diagnosis is given

if vid not in voorschriften and start + single_day < orderstart or start > stop: continue

merged = False

# voorschrift is within range so is included

if start_stop_times[0] <= start + single_day and start <= start_stop_times[1] + single_day:

merged = True

# voorschrift ends within range so is included

elif start_stop_times[0] <= stop + single_day and stop <= start_stop_times[1] + single_day:

merged = True

# voorschrift starts within range so is included

elif start_stop_times[0] + single_day >= start and stop >= start_stop_times[1] - single_day:

merged = True

# There is overlap, so we need to merge

if merged:

# Merge start

start_stop_times[0] = min([start, orderstart, start_stop_times[0]])

# Merge stop

start_stop_times[1] = max([stop, orderstop, start_stop_times[1]])

# Add vid

start_stop_times[2].append(vid)

# add label if voorschrift is longer than 28 days

if (stop - start).days > 28:

lange_voorschrift = 1

if len(start_stop_times) > 0:

matched_all_vs.append([pid, id, vraag, antwoord, ab, start_stop_times[0], start_stop_times[1], orderstart, lange_voorschrift])

## Add voorschift to candidates for merging

og_vs_dict[pid][ab].append([start_stop_times[0], start_stop_times[1], id])

voorschriften.add(id)

else:

matched_all_vs.append([pid, id, vraag, antwoord, "", "", "", orderstart, lange_voorschrift])

print("matched_all_vs: ", len(matched_all_vs))

# create final table

unique_start = {}

for vs in matched_all_vs:

pid = vs[0]

if vs[5] != "":

if pid not in unique_start:

unique_start[pid] = [vs[5]]

else:

unique_start[pid].append(vs[5])

print("unique_start: ", len(unique_start))

# sort and filter unique start dates

ranked_start = {}

for pid in unique_start:

if pid not in ranked_start:

ranked_start[pid] = {}

sorted_filtered_start = sorted(list(set(unique_start[pid])))

unique_count = 0

for start in sorted_filtered_start:

ranked_start[pid][start] = unique_count

unique_count += 1

print("ranked_start: ", len(ranked_start))

# Add unique start to result and create final table

result = []

for vs in matched_all_vs:

pid = vs[0]

start = vs[5]

if start != "":

vs.insert(len(vs), ranked_start[pid][start])

result.append(vs)

else:

vs.insert(len(vs), "")

result.append(vs)

print("result: ", len(result))

result = pd.DataFrame(result)

result.columns = ["Pseudo_id", "Voorschrift_id", "Vraag", "Antwoord", "ATC_naam", "Startdatum", "Stopdatum", "OrderStartDatumtijd", "langer dan 28 dagen Voorschrift", "KuurID"]

**Additional file 3. Error rate in validated sample of courses, broken down per infection**

|  | **Incorrect Registered indication, n (%)** | **Incorrect LOT based on prescriptions, n (%)** | **Incorrect LOT based on clinical notes, n (%)** |
| --- | --- | --- | --- |
| **Intra-abdominal infection** | 6/84 (7.4) | 2/84 (2.4) | 22/84 (26.2) |
| **Respiratory tract infections (specified)** | 23/112 (20.5) | 2/112 (1.8) | 17/112 (15.2) |
| RTI-CAP-m | 7/42 (16.7) | 0/42 (0.0) | 7/42 (16.7) |
| RTI-CAP-s | 8/12 (66.7) | 0/12 (0.0) | 0/12 (0.0) |
| RTI-HAP | 1/11 (9.1) | 1/11 (9.1) | 1/11 (9.1) |
| RTI-COPD | 3/13 (23.1) | 1/13 (7.7) | 4/13 (30.8) |
| RTI Aspiration | 0/13 (0.0) | 0/13 (0.0) | 0/13 (0.0) |
| RTI abscess/empyema | 0/5 (0.0) | 0/5 (0.0) | 0/5 (0.0) |
| RTI-other (not specified) | 4/16 (25.0) | 0/16 (0.0) | 5/16 (31.3) |
| **Respiratory tract infection (overall error*)** | 5/112 (4.5) |  |  |
| **Urinary tract infections (specified)** | 38/131 (29.0) | 0/131 (0.0) | 30/131 (22.9) |
| UTI-cystitis | 24/60 (40.0) | 0/60 (0.0) | 15/60 (25.0) |
| UTI-complicated | 1/33 (3.0) | 0/33 (0.0) | 6/33 (18.2) |
| UTI-kidney transplant | 0/12 (0.0) | 0/12 (0.0) | 3/12 (25.0) |
| UTI-Catheter related | 2/7 (28.6) | 0/7 (0.0) | 2/7 (28.6) |
| UTI-cyst | 0/1 (0.0) | 0/1 (0.0) | 0/1 (0.0) |
| UTI-prostatitis | 4/4 (100.0) | 0/4 (0.0) | 1/4 (25.0) |
| UTI-other (not specified) | 7/14 (50.0) | 0/14 (0.0) | 3/14 (21.4) |
| **Urinary tract infection (overall error*)** | 10/131 (7.6) |  |  |
| **Total of other infections** | 44/202 (21.8) | 4/202 (2.0) | 42/202 (20.8) |
| Bone or joint infection | 5/29 (17.2) | 2/29 (6.9) | 11/29 (37.9) |
| CNS infection | 0/5 (0.0) | 0/5 (0.0) | 1/5 (0.2) |
| E.N.T. or oral and maxillofacial | 1/15 (6.7) | 0/15 (0.0) | 5/15 (33.3) |
| Endovascular infection | 0/2 (0.0) | 0/2 (0.0) | 1/2 (50.0) |
| Eye infection | 0/2 (0.0) | 0/2 (0.0) | 1/2 (50.0) |
| Febrile neutropenia | 0/7 (0.0) | 0/7 (0.0) | 0/7 (0.0) |
| Fungal infection | 2/3 (66.7) | 0/3 (0.0) | 2/3 (66.7) |
| Gastro-enteritis | 3/6 (50.0) | 0/6 (0.0) | 1/6 (16.7) |
| Gynecological infection | 3/17 (17.6) | 0/17 (0.0) | 1/17 (5.9) |
| CVL infection | 1/9 (11.1) | 0/9 (0.0) | 4/9 (44.4) |
| Mediastinitis | 1/5 (20.0) | 0/5 (0.0) | 2/5 (40.0) |
| SAB | 0/5 (0.0) | 0/5 (0.0) | 2/5 (40.0) |
| Sepsis e.c.i. | 23/52 (44.2) | 2/52 (3.8) | 4/52 (7.7) |
| skin or soft tissue infection | 5/45 (11.1) | 0/45 (0.0) | 7/45 (15.6) |
| **Total of validated infections** | 111/529 (21.0) | 8/529 (1.5) | 111/529 (21.0) |

LOT = length of treatment, RTI = respiratory tract infection, CAP-m = mild-to-moderate severe community-acquired pneumonia (PSI 1-2), CAP-s = severe community-acquired pneumonia (PSI 3-5), HAP = hospital-acquired pneumonia, COPD = chronic obstructive pulmonary disease, UTI = urinary tract infection, CNS = central nervous system, E.N.T. = ear, nose, and throat, CVL = central venous line, SAB = *Staphylococcus aureus* bacteremia. *Overall error: the registered indication was assigned to the wrong site of infection.
